# Energy conservation under extreme energy limitation: the role of cytochromes and quinones in acetogenic bacteria

**DOI:** 10.1007/s00792-021-01241-0

**Published:** 2021-09-04

**Authors:** Florian P. Rosenbaum, Volker Müller

**Affiliations:** Molecular Microbiology and Bioenergetics, Institute of Molecular Biosciences, Johann Wolfgang Goethe University, Max-von-Laue-Str. 9, 60438 Frankfurt, Germany

**Keywords:** Anaerobes, Electron transport chain, Extremophiles, Thermophiles, Wood–Ljungdahl pathway

## Abstract

Acetogenic bacteria are a polyphyletic group of organisms that fix carbon dioxide under anaerobic, non-phototrophic conditions by reduction of two mol of CO_2_ to acetyl-CoA via the Wood–Ljungdahl pathway. This pathway also allows for lithotrophic growth with H_2_ as electron donor and this pathway is considered to be one of the oldest, if not the oldest metabolic pathway on Earth for CO_2_ reduction, since it is coupled to the synthesis of ATP. How ATP is synthesized has been an enigma for decades, but in the last decade two ferredoxin-dependent respiratory chains were discovered. Those respiratory chains comprise of a cytochrome-free, ferredoxin-dependent respiratory enzyme complex, which is either the Rnf or Ech complex. However, it was discovered already 50 years ago that some acetogens contain cytochromes and quinones, but their role had only a shadowy existence. Here, we review the literature on the characterization of cytochromes and quinones in acetogens and present a hypothesis that they may function in electron transport chains in addition to Rnf and Ech.

## Introduction

Acetogenic bacteria are a polyphyletic group of strict anaerobes that are found in every possible ecosystem such as soil and water sediments or the animal intestine but also in hot, alkaline, acidic or saline environments (Drake et al. 2008). This universal distribution reflects their essential role in anaerobic food webs for they funnel carbon from various substrates such as sugars, aldehydes, carbonic acids, secondary alcohols but also C1 substrates and especially O-methyl, N-methyl and S-methyl groups to acetate, which is then finally converted by methanogenic archaea to methane (Daniel et al. 1988; Schink 1994; Drake et al. 2008; Schuchmann and Müller 2016). Oxidation under anoxic conditions of many of these substrates to carbon dioxide and hydrogen gas is highly endergonic, and thus does not allow microbes to grow on them unless, for example, the oxidation is coupled to the reduction of an external electron acceptor such as sulfate or carbon dioxide.

**Fig. 1:**
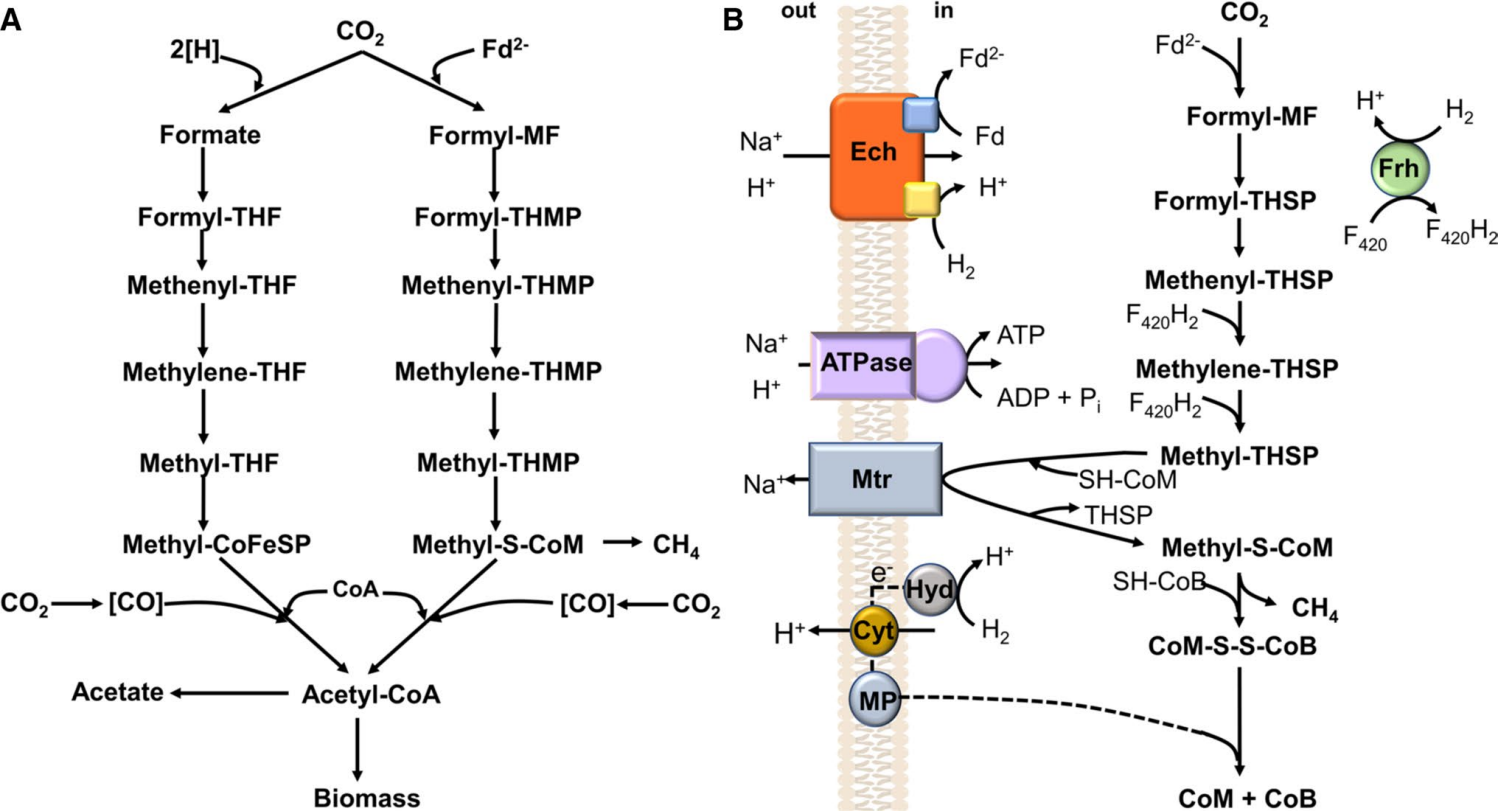
The Wood–Ljungdahl pathway of acetyl-CoA formation from CO_2_ in acetogenic bacteria (**A**, left) and methanogenic archaea (**A**, right). In the anabolic route, acetyl-CoA is further converted to pyruvate and from there into the different biosynthetic routes. In the catabolic routes, acetate and methane are the end products (modified after Ljungdahl 1986; Welte and Deppenmeier 2014). **B** The carbon and electron flow in methanogenesis coupled to ATP synthesis by a chemiosmotic mechanism in cytochrome-containing *Methanosarcina* species (modified after Welte and Deppenmeier 2014). The ion specificity of the ion-translocating enzymes is shown, but the stoichiometry is not indicated since it is unknown. [H], reducing equivalent, one electron; cyt, cytochrome; MP, methanophenazine; F_420_, coenzyme F_420_; F_420_H_2_, reduced coenzyme F_420_; Frh, coenzyme F_420_ reducing hydrogenase; Mtr, methyl-THSP:CoM methyltransferase

Carbon dioxide reduction with hydrogen as electron donor is a hallmark of methanogenic archaea and acetogenic bacteria (Thauer et al. 2010; Drake et al. 2008). Both use the acetyl-CoA or Wood–Ljungdahl pathway (WLP) for the synthesis of a C2 unit, acetyl-CoA, from carbon dioxide (Fig. 1), which is further reductively carboxylated to pyruvate and from there funneled into the known routes for the biosynthesis of cellular monomers and polymers (Blamey and Adams 1993). The WLP is a two-branched linear pathway in which two molecules of CO_2_ are converted to acetyl-CoA (Ljungdahl 1986; Wood et al. 1986). In the methyl branch, the methyl group of acetyl-CoA is generated from CO_2_. The first step is the reduction of CO_2_ to a formyl group and its activation by adding it to a C1-carrying cofactor. In acetogens, this is done by two subsequent reactions, a formate dehydrogenase reduces CO_2_ to formate, which is then bound to the C1 carrier tetrahydrofolate (THF) by the enzyme formyl-THF synthetase, at the expense of ATP hydrolysis (Himes and Harmony 1973; Moon et al. 2021). In methanogens, the formyl-methanofuran dehydrogenase reduces CO_2_ and binds the carbonyl group to the C1 carrier methanofuran (MF) yielding formyl-methanofuran (formyl-MF) (Leigh et al. 1985). Next, the formyl group is transferred to the other C1 carrier tetrahydromethanopterin (or tetrahydrosarcinapterin) and from there, the further conversion of the carbonyl group is similar in acetogens and methanogens (Kengen et al. 1988; van Beelen et al. 1984). From formyl-THF/THMP water is split off, and the resulting methenyl group is reduced via the methylene reductase to a methyl group (Clark and Ljungdahl 1982; Moore et al. 1974). In the carbonyl branch, the carbonyl group of acetyl-CoA is formed from another mol of CO_2_ by action of the CO dehydrogenase/acetyl-CoA synthase (CODH/ACS), the key enzyme of the pathway (Pezacka and Wood 1984; Raybuck et al. 1988). In their catabolism, the two microbial groups use different routes. In methanogens, the methyl group from methyl-THMP/THSP is transferred by a methyltransferase to coenzyme M (2-mercaptoethanesulfonate) yielding methyl-CoM. The methyltransferase is a membrane-integral protein and uses the free energy of the methyltransferase reaction to expel sodium ions from the cells, thus generating an electrochemical sodium ion potential across the membrane that then drives the synthesis of ATP via an archaeal A_1_A_O_ ATP synthase (Deppenmeier et al. 1996; Gottschalk and Thauer 2001; Müller et al. 1988a, 1999). In contrast to electron transport phosphorylation, this type of energy conservation is called methyltransfer-driven phosphorylation; both types are subsummarized under the term “chemiosmotic energy conservation” (Hess et al. 2013). In the next step, coenzyme B (7-mercaptoheptanoylthreoninephosphate) attacks the methyl group of methyl-CoM, methane is produced, and a disulfide of CoM and CoB is formed, the heterodisulfide (Rouvière and Wolfe 1988). The reduction of the heterodisulfide with H_2_ is an exergonic reaction. And now we come to the roles of cytochromes. Some methanogens such as the *Methanosarcinaea* have a cytochrome-containing, proton-translocating electron transport chain to reduce the heterodisulfide (Deppenmeier et al. 1996). This electron transport chain generates an electrochemical proton potential across the cytoplasmic membrane that drives the synthesis of ATP, again by an A_1_A_O_ ATP synthase (Deppenmeier and Müller 2008). Most of the methanogens do not have cytochromes, they employ electron bifurcation to use the exergonic reduction of the heterodisulfide to lift up the energetic level of the electron coming from hydrogen so that it reaches the energy level of ferredoxin (Thauer et al. 2008). Now, we have to turn back to the first reaction that is highly endergonic with H_2_ as electron donor but feasible with reduced ferredoxin. In the cytochrome-free methanogens, the reduced ferredoxin generated by the electron-bifurcating heterodisulfide reaction is directly used for CO_2_ reduction to formyl-MF, whereas in the cytochrome-containing methanogens, the electrochemical proton potential established in the heterodisulfide reaction is used to drive reverse electron transport from hydrogen to ferredoxin catalyzed by a membrane-integral, proton-translocating, energy-converting hydrogenase (Ech) (Lie et al. 2012). So, cytochrome-containing methanogens take a little detour to reduce ferredoxin, which is required for the first reaction. This detour makes them much more flexible in that the electrochemical proton potential can, of course, also be used for other membrane-associated cellular work that has to be done by every living cell, such as osmotic work (substrate transport) and chemical work (ATP synthesis). Remarkably, the flagellum of archaea is unique, it is not using the electrochemical proton/sodium ion potential as driving force for mechanical work but ATP hydrolysis (Streif et al. 2008).

The review of the history of energy conservation in methanogens taught us the following lessons: (1) there is no substrate level phosphorylation in the WLP leading from CO_2_ to a methyl group, (2) one of the enzymes of the WLP, the methyltransferase, is a “respiratory” enzyme, (3) cytochromes were before seen as the hallmark of aerobes and their respiratory mechanisms. Their discovery in methanogens immediately led to the hypothesis that they must be involved in some sort of respiratory chain. (4) Most methanogens do not have cytochromes and do conserve energy in form of ATP by methyltransfer-driven phosphorylation only.

## Energy conservation in acetogens

Having the history of energy conservation in methanogens in mind, we can now move on to acetogenic bacteria. During growth on H_2_ + CO_2_, carbon dioxide is reduced to methyl-THF (Fig. 1). The methyl group is then transferred to a corrinoid/iron sulfur protein (CoFeSP) from which it is transferred to the CODH/ACS where it condenses with CoA and CO to acetyl-CoA (Diekert and Ritter 1982; Drake et al. 1980; Hu et al. 1982). Phosphotransacetylase catalyzes the formation of acetyl phosphate and acetate kinase the formation of acetate and ATP. The overall free energy change of the reaction (Eq. 1):

1 $${\text{2 CO}}_{{2}} + {\text{ 4 H}}_{{2}} \, \to \,{\text{ CH}}_{{3}} {\text{COO}}^{ - } \, + \,{\text{H}}^{ + } \, + \,{\text{2 H}}_{{2}} {\text{O}}\;\Delta {\text{G}}_{0}^{\prime } \, = \, - {95}\,{\text{kJ/mol,}}$$

is only enough to make only around 1.5 ATP under standard conditions (Schuchmann and Müller 2014). However, if we take into calculation the actual hydrogen partial pressures that have been determined in the environments, which is less than 1 µM, we end up with approximately 0.3 mol ATP/mol acetate (Conrad et al. 1986). This is life at the thermodynamic edge and the question was for a long time how this pathway is coupled to the net synthesis of ATP (Müller 2003). One ATP is gained in the acetate kinase reaction, but one ATP has to be invested in the activation of formate; therefore, the ATP gain by substrate level phosphorylation is zero and an additional mechanism of energy conservation must exist. Having described that the discovery of cytochromes in a species is ultimately seen as evidence for an energy-conserving respiratory chain, it is not surprising that the discovery of cytochromes in one acetogen excited the entire field. The discovery of *b*-type cytochromes in *Moorella thermoacetica* (formerly *Clostridium thermoaceticum*) in 1975 by Gottwald et al. was immediately taken as indication for cytochrome-dependent electron transport chain as part of a chemiosmotic mechanism of ATP synthesis and extrapolated to the entire group of acetogens (Gottwald et al. 1975; Hugenholtz and Ljungdahl 1990). Later, reduction of cytochromes as well as the concomitant generation of a membrane potential in vesicles of *Moorella thermoautotrophica* (formerly *Clostridium thermoautotrophicum)* was detected but the physiological processes involved remained enigmatic (Hugenholtz et al. 1987).

**Fig. 2:**
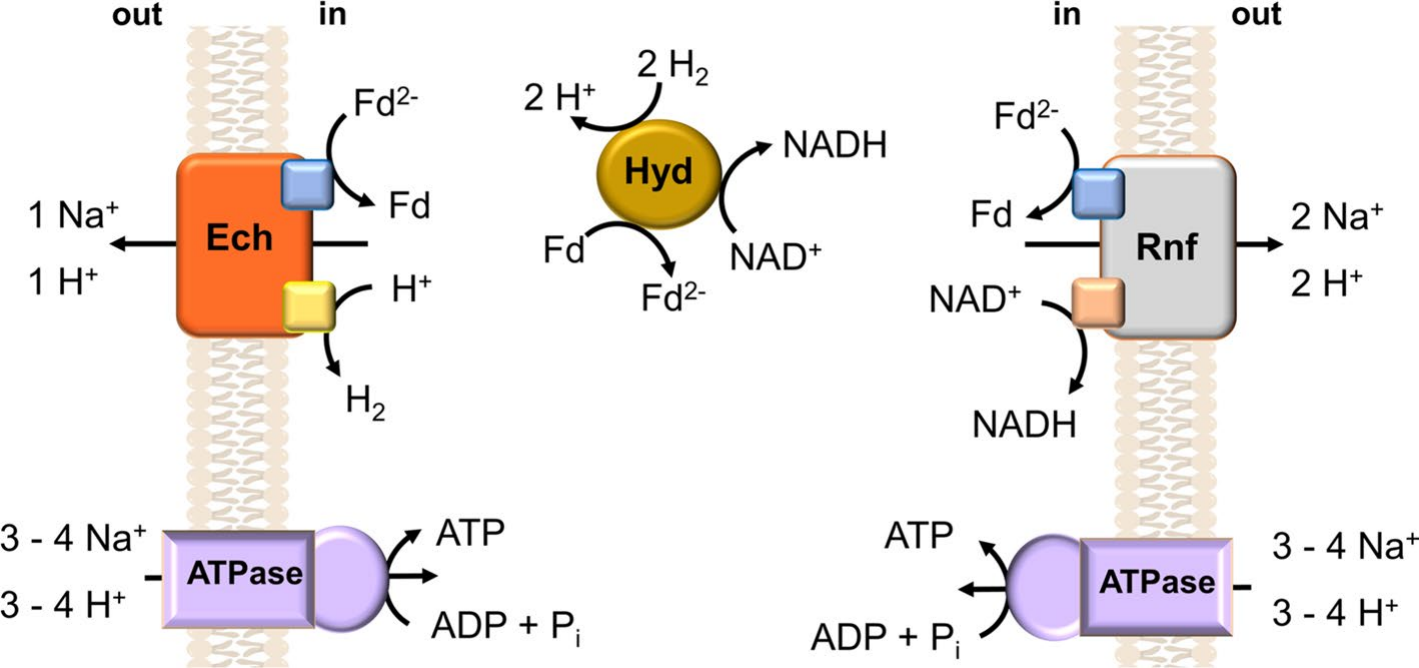
Energy conservation by a chemiosmotic mechanism in acetogenic bacteria. Depicted is an Ech- (left) and a Rnf-containing respiratory chain (right). An electron-bifurcating, ferredoxin and NAD-reducing hydrogenase provides reduced ferredoxin as fuel for both electron transport chains (Schuchmann and Müller 2012). In principle Rnf, Ech and ATP synthase can use Na^+^ or H^+^ as coupling ion. This has been experimentally verified for Rnf (Na^+^ in *A. woodii* by Hess et al. 2013, H^+^ in *C. ljungdahlii* by Tremblay et al. 2012), Ech (Na^+^ and H^+^ in *T. kivui* by Schoelmerich and Müller 2019) and ATP synthase (Na^+^ in *A. woodii* by Reidlinger and Müller 1994; H^+^ in *M. thermoacetica* by Das and Ljungdahl 1997). See Table 1. for ion specificities of ATP synthases in acetogens. Ech is indicated to pump one ion per two electrons, Rnf two ions per two electrons. The stoichiometry is based on thermodynamic considerations

The beauty of microbiology is mirrored in the beauty of microbial diversity. Today, 23 genera of acetogens are described and acetogenic bacteria are spread over the entire phylogenetic tree (Drake et al. 2008; Müller and Frerichs 2013). Some tribes have only acetogens, other have other metabolic types as well. Therefore, as with the methanogens, only a few acetogens do have membrane-bound electron carriers such as cytochromes or quinones (Table 1), the majority does not have cytochromes or quinones! Indeed, the first conclusive evidence how acetogens conserve energy comes from a study with *Acetobacterium woodii*, a species that does neither have cytochromes nor quinones. First, although it had been postulated based on the finding of a “respiratory” methyltransferase in methanogens that a methyltransferase of the WLP in acetogens is the chemiosmotic coupling site, this could be ruled out with certainty for *A. woodii* and for any other sequenced acetogen (Müller 2003; Schuchmann and Müller 2014). Second, the energy-conserving site is not in the WLP per se, but in the electron pathway leading to the reduction of electron carriers required for the WLP. Membranes of *A. woodii* have a novel respiratory electron transport complex that catalyzes ferredoxin:NAD oxidoreductase activity, the Rnf complex, where Rnf stands for “Rhodobacter nitrogen fixation” (Fig. 2) (Imkamp et al. 2007; Biegel and Müller 2010; Biegel et al. 2011; Hess et al. 2013, 2016). The enzyme is reversibly coupled to the electrochemical ion potential across the membrane and pumps out Na^+^ when electron flow is exergonic, that is from reduced ferredoxin to NAD, whereas the endergonic backward reaction is energetically driven by Na^+^ influx along the electrical field (Hess et al. 2013). Again, the beauty of metabolic diversity comes into our way, Rnf complexes from some acetogens use Na^+^, others use H^+^ instead (Table 1). This is not surprising considering that the change of only two amino acids out of roughly 7000 changes the substrate specificity from H^+^ to Na^+^ in ATP synthases (Müller and Grüber 2003; Grüber et al. 2014). As a side note it is important to realize that acetogens can thrive on energy poor substrates that can only be used to reduce NAD; then, the Rnf complex is used to provide the cell with reduced ferredoxin for biosynthetic reactions (Bertsch et al. 2016; Westphal et al. 2018).

**Table 1:** Presence of Rnf, Ech, Ion dependence of the ATP synthase, presence of cytochromes, quinones, HdrABCMvhD and Fix complexes in acetogens

Organism	Rnf	Ech	ATP synthase	Cyt^1^	Quinon^2^	Hdr	Fix	Remarks
*Acetobacterium woodii* DSM 1030	+	−	Na^+^	−	−	−	−	
*Acetobacterium bakii* DSM 8239	+	*	Na^+^	−	−	−	−	Ech-like complex*
*Acetobacterium dehalogenans* DSM 11527	+	−	Na^+^	−	−	−	−	
*Acetobacterium wieringae* DSM 1911	+	−	Na^+^	−	−	−	−	
*Acetoanaerobium noterae* ATCC 35199	+	−	Na^+^	−	−	−	−	
*Acetoanaerobium sticklandii* DSM 519	+	−	Na^+^	−	−	−	−	
*Acetohalobium arabaticum* DSM 5501	+	*	Na^+^	−	+ ^3^	−	−	Ech-like complex*
*Acetonema longum *DSM 6540	−	+	_H_ ^+^	−	+ ^3^	−	−	
*Acetitomaculum ruminis *DSM 5522	+	−	Na^+^	−	−	−	−	
*Alkalibaculum bacchi *DSM 22112	+	−	Na^+^	−	−	−	−	
*Blautia coccoides *DSM 935	+	−	_H_ ^+^	−	−	+	−	
*Blautia hydrogenotrophica* DSM 10507	+	−	_H_ ^+^	−	−	−	−	
*Blautia luti*	+	−	_H_ ^+^	−	−	+	−	
*Blautia producta DSM 2950*	+	−	_H_ ^+^	−	−	+	−	
*Blautia schinkii *10518	+	−	_H_ +	−	−	−	−	
*Blautia wexlerae *19850	+	−	_H_ ^+ ^	−	−	+	−	
*Clostridium aceticum *DSM 1496	+	−	_Na_ ^+^	c	−	−	−	Cyt. and nitrite reductase in one cluster
*Clostridium acetobutylicum *DSM 1732	+	−	_H+_	−	−	−	−	
*Clostridium autoethanogenum* DSM 10061	+	−	_H_ ^+^	−	−	−	#	Fix-like complex^#^
*Clostridium carboxidivorans* DSM 15243	+	*	_H_ ^+^	−	−	−	−	Ech-like complex*
*Clostridium coskatii *ATCC PTA-10522	+	−	_H_ ^+^	−	−	−	#	Fix-like complex^#^
*Clostridium difficile* 630 DSM 27543	+	−	Na^+^	−	−	−	−	
*Clostridium drakei *DSM 12750	+	−	_H_ ^+^	−	−	−	−	
*Clostridium formicaceticum* DSM 92	+	−	Na^+^	c	−	−	−	Cyt. and nitrite reductase in one cluster
*Clostridium ljungdahlii *DSM 13528	+	−	_H_ ^+^	−	−	−	#	Fix-like complex^#^
*Clostridium magnum *DSM 2767	+	−	_H_ ^+^	−	−	−	#	Fix-like complex^#^

Organism	Rnf	Ech	ATP synthase	Cyt	Quinon	Hdr	Fix	Additional Information
*Clostridium methoxybenzovorans* DSM 12182	+	−	_H_ ^+^	−	−	−	−	
*Clostridium ragsdalei* DSM 15248	+	−	_H_ ^+^	−	−	−	−	
*Clostridium scatologenes* DSM 757	+	*	_H_ ^+^	−	−	−	−	Ech-like complex*
*Clostridium *sp*.* AWRP	+	−	_H_ ^+^	−	−	−	−	
*Clostridium *sp*.* P21	+	−	_H_ ^+^	−	−	−	−	
*Clostridium ultunense* DSM 10521	+	−	Na^+^	−	−	−	−	
*Eubacterium aggregans* SR 12	+	−	Na^+^	−	−	+	−	
*Eubacterium limosum* KIST612	+	−	Na^+^	−	−	−	−	
*Holophaga foetida* 6591	−	*	_H_ ^+^	−	−	−	−	Ech-like complex*
*Moorella glycerini* NMP	−	+	_H_ ^+^	b	MQ	+	+	
*Moorella humiferrea* DSM 23265	−	*	_H_ ^+^	b	MQ	+	+	Ech-like complex*
*Moorella mulderi* DSM 14980	−	+	_H_ ^+^	b	MQ	+	+	
*Moorella thermoacetica *DSM 521	−	+	_H_ ^+^	b	MQ	+	+	
*Moorella thermoautotrophica*	−	+	_H_ ^+ ^	b	MQ	+	+	
*Sporomusa acidovorans* DSM 3132	+	−	_H_ ^+^	b/c	UQ	+	+	
*Sporomusa aerivorans *DSM 13326	+	−	_H_ ^+^	b/c	UQ	+	+	
*Sporomusa malonica *DSM 5090	+	−	_H_ ^+^	b/c	UQ	+	+	
*Sporomusa ovata *DSM 2662	+	*	_H_ ^+^	b/c	UQ	+	+	Ech-like complex fix-complex split into two clusters*
*Sporomusa sphaeroides* Strain E	+	−	_H_ ^+^	b/c	UQ	+	+	
*Sporomusa silvacetica* DSM 10669	+	−	_H_ ^+^	b/c	UQ	+	+	
*Sporomusa termitida*	+	−	_H_^+^	b/c	UQ	+	+	
*Thermoanaerobacter kivui* DSM 2030	−	+	_H_ ^+^	−	−	−	−	
*Thermacetogenium phaeum* DSM 12270	−	+	_H_ ^+^	−	MQ	+	+	
*Tindallia californiensis* APO	−	+	_Na_ ^+^	−	−	+	−	
*Treponema azotonutricium* DSM 13862	+	−	_H_ +	−	−	−	−	

^1^”c” or “b” denotes the type of cytochrome

^2^*MQ* menaquinone, *UQ* ubiquinone

^3^not further characterized

^*^”Ech-like complex” denotes for the presence of genes that encode proteins with similarity to some Ech subunits, but not a full complex

^#^”Fix-like complex” denotes for a complex present in quinone-free organisms; therefore, its function cannot be as described in quinone-containing organisms

Again, we are hit by the beauty of metabolic diversity. Inspection of genome sequences revealed that the Rnf complex is not universally found in acetogens and nearly 10 years after the discovery of the Rnf complex, a second respiratory enzyme was found in acetogens, the Ech complex (Fig. 2) (Schoelmerich and Müller 2019). The Ech also uses reduced ferredoxin as electron donor for an enzyme-bound electron transport chain, but protons, not NAD, as acceptor, leading to the production of hydrogen gas as end product of this respiration (Schoelmerich and Müller 2019, 2020; Welte et al. 2010). As of today, acetogens have either Rnf or Ech, not both, in one cell (Table 1), although some non-acetogenic rumen bacteria have been reported to have Rnf and Ech complexes operative in one cell (Hackmann and Firkins 2015; Schoelmerich et al. 2020). Anyway, the important message is that every acetogen analyzed or sequenced so far possesses either Rnf or Ech (Fig. 2). Therefore, it is justified to energetically classify the acetogens in the groups of Rnf and Ech acetogens (Schuchmann and Müller 2014). Since Rnf and Ech are electron transport-driven ion pumps operating by conformational changes, both complexes can, in principle, translocate Na^+^ or H^+^, based on the nature of the ion-binding pocket. The same change in ion specificity is found in ATP synthases, the flagellar motor, ion-coupled secondary transporter or the transhydrogenase (Homma et al. 1985; Kawagishi et al. 1996; Müller and Grüber 2003; Khafizov et al. 2012; Luoto et al. 2013).

## Cytochromes and quinones in acetogens

After having worked out that every acetogen known to date has a ferredoxin-dependent respiratory enzyme that does not involve cytochromes the question of course is: what is the role of the cytochromes? A solution to this question may come from the analysis of the distribution of cytochromes, their different nature and the physiological activities of the organisms cytochromes have been found in. The picture is complicated by the fact that many acetogens can reduce a number of alternative substrates such as pyruvate, fumarate, aromatic acrylates, inorganic sulfur compounds and nitrate or nitrite in addition to CO_2_ (Dorn et al. 1978; Bache and Pfennig 1981; Tschech and Pfennig 1984; Beaty and Ljungdahl 1990; Matthies et al. 1993; Seifritz et al. 1993, 2003; Fröstl et al. 1996; Misoph et al. 1996; Misoph and Drake 1996; Arendsen et al. 1999; Hattori et al. 2000).

*c*-type cytochromes such as cytochrome *c*_*3*_ ($E_{0}^{\prime }$ = −200 mV) are typical constituents of respiratory chains leading to the reduction of nitrate (Thauer et al. 1977). Some *Sporomusa* and *Clostridia* species can reduce nitrate, which may involve cytochrome c as electron donor. Furthermore, *C. ljungdahlii* can co-utilize CO_2_ and nitrate with a reductase which is not associated to cytochrome c. This co-utilization of CO_2_ and nitrate enhanced biomass formation from H_2_ + CO_2_ (Emerson et al. 2019). Mechanistically, it is not known whether nitrate reduction is coupled to energy conservation for example via a nitrate-dependent ion-motive respiratory chain or whether the presence of nitrate redirects electrons to the Rnf- and Ech complexes thus providing more fuel for chemiosmotic ATP synthesis (Seifritz et al. 1993, 2002). A proteome study revealed that the genes for the biosynthesis of cytochrome *c* were upregulated together with the genes encoding nitrate and nitrite reductase specifically in the presence of nitrate (Visser et al. 2016). Furthermore, a similar circumstance can be suggested for the acetogens *Clostridium aceticum* and *Clostridium formicoaceticum* since genes involved for the cytochrome *c* synthesis are clustered with a nitrate reductase (Table 1). Although this is only circumstantial evidence it is very plausible that cytochrome *c* is involved in electron transport towards nitrate and maybe other alternative electron acceptors as well. Intercellular electron transfer may also be possible, but this has not been addressed in acetogens.

*b*-type cytochromes such as cytochrome *b*_*559*_ or *b*_*554*_ (E_0_′ = − 200 and − 48 mV) are known as membrane anchors of dehydrogenases and hydrogenases that transfer electrons via the membrane-bound electron carriers (Das et al. 1989; Dobrindt and Blaut 1996; Kröger et al. 2002). In *M. thermoacetica*, nitrate represses acetate formation as terminal electron acceptor through the WLP and in one study, the activity of the enzymes of the WLP was not affected, whereas in another it was found that the synthesis of the WLP enzymes was downregulated by nitrate, including the synthesis of the *b*-type cytochrome (Arendsen et al. 1999; Fröstl et al. 1996). This would argue for the *b*-type cytochrome being involved in electron transfer to the WLP enzymes.

## Hypothesis: membrane-bound, cytochrome- and/or quinone-dependent electron transport chains in acetogens

**Fig. 3:**
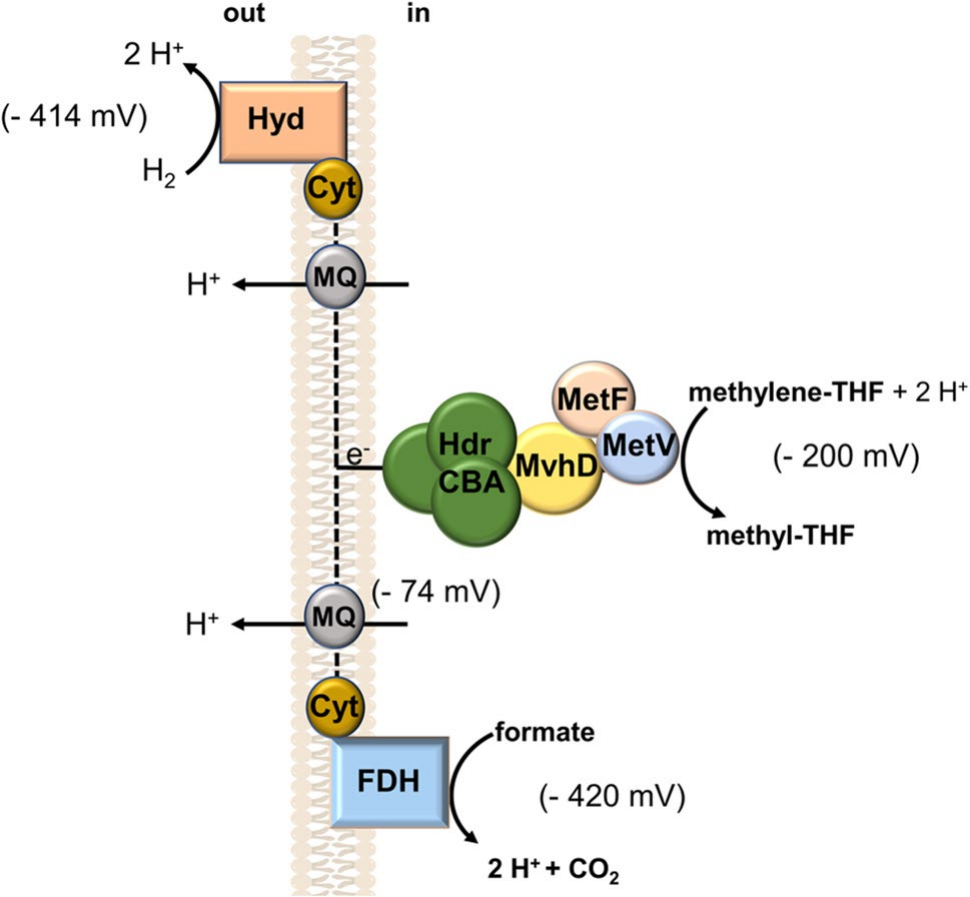
Hypothetical cytochrome- and menaquinone-containing energy-conserving electron transport chains. In this example, the MetFVHdrABCMvhD complex is not indicated to bifurcate electrons to two acceptors. Please note that proton translocation is related to oxidation/reduction of MQ and requires a membrane-integrated protein to catalyze this reaction. Hyd, hydrogenase; FDH, formate dehydrogenase; Cyt, cytochrome; MQ, menaquinone; MetFV, methylene-THF reductase; HdrABCmvhD, a hypothetical linker of the MetFV methylene-THF reductase to a component in the cytoplasmic membrane

If we consider the argument again that *b*-type cytochromes are membrane anchors for dehydrogenases and hydrogenases what could be the donors and acceptors of such electron transport chain? Of course, they could be very different given the beauty of diversity in electron donors and acceptors used by acetogens. But if we, for now, focus on the WLP, reduction of methylene-THF to methyl-THF (E_0_’ = -200 mV) has a very positive redox potential and reduction of methylene-THF with NADH as reductant is the most exergonic reaction of the pathway (Wohlfarth and Diekert 1991). Whether or not this reaction is energy conserving has not been proven, despite the fact that this was already postulated nearly 45 years ago (Thauer et al. 1977). The most conclusive evidence that the *b*-type cytochromes are involved in electron transfer to the WLP was presented by Kamlage and Blaut, already in 1993 (Kamlage and Blaut 1993). By showing that a cytochrome *b*-deficient mutant was no longer able to oxidize methyl groups to CO_2_ or reduce CO_2_ to the level of a methyl group, they laid the foundation for the hypothesis that *b*-type cytochromes are involved as electron carrier in electron transfer to methylene-THF (MTHF) (Kamlage and Blaut 1993). Later on, the group of Michael Blaut isolated a membrane-bound hydrogenase that had a cytochrome *b*-containing subunit (Dobrindt and Blaut 1996). With that discovery it was plausible to assume a periplasmic hydrogenase that oxidizes hydrogen at the outside, thus generating scalar protons. The electron is then passed via cytochrome *b* to the acceptor, methylene-THF reductase (MTHFR) (Fig. 3). ATP is synthesized driven by the electrochemical proton potential generated by the scalar protons. It should be mentioned that such hydrogenase-dependent, energy-conserving electron transport chains are widespread in the anaerobic world (Kröger et al. 2002).

An involvement of cytochromes in the MTHFR reaction was also very recently speculated by Keller et al. (2019) for the thermophile *Thermacetogenium phaeum* (Keller et al. 2019). This acetogen can grow on formate that is converted to acetate via the WLP. From a detailed proteomic and enzymatic study, it was suggested that two mol of formate are oxidized by a soluble formate dehydrogenase to CO_2_ to provide NADH and ferredoxin for the methylene-THF dehydrogenase and CODH/ACS, respectively. The third mole of formate is suggested to be oxidized by a membrane-bound formate dehydrogenase/quinone oxidoreductase to reduce menaquinone-7 (E_0_’ = -74 mV). The final acceptor is supposed to be the MTHFR (Fig. 3). A similar system was proposed on theoretical grounds for *M. thermoacetica* (Mock et al. 2014). Since there is no indication that the formate dehydrogenase and the MTHFR are periplasmic, energy conservation must occur by vectorial proton transport coupled to this reaction.

The WLP is reversible and is used by *T. phaeum* for the oxidation of acetate to formate/H_2_ + CO_2_ or by *S. ovata* and *S. sphaeroides* for the oxidation of methanol to CO_2_ and H_2_. Therefore, it is mandatory that the before mentioned electron transport chains are reversible. MTHF oxidation with NAD reduction is endergonic and the energy to overcome the energy barrier is provided in this example by reverse electron transport. Mutant studies with *S. sphaeroides* are in fact in line with this hypothesis as well as proteome studies in *T. phaeum* (Kamlage and Blaut 1993; Keller et al. 2019). In the latter, acetate is oxidized to formate which is then transferred to a syntrophic partner, to make the overall acetate oxidation energetically feasible. However, proteome analyses revealed evidence for a different formate dehydrogenase present in the periplasm thus producing a scalar proton gradient.

**Fig. 4:**
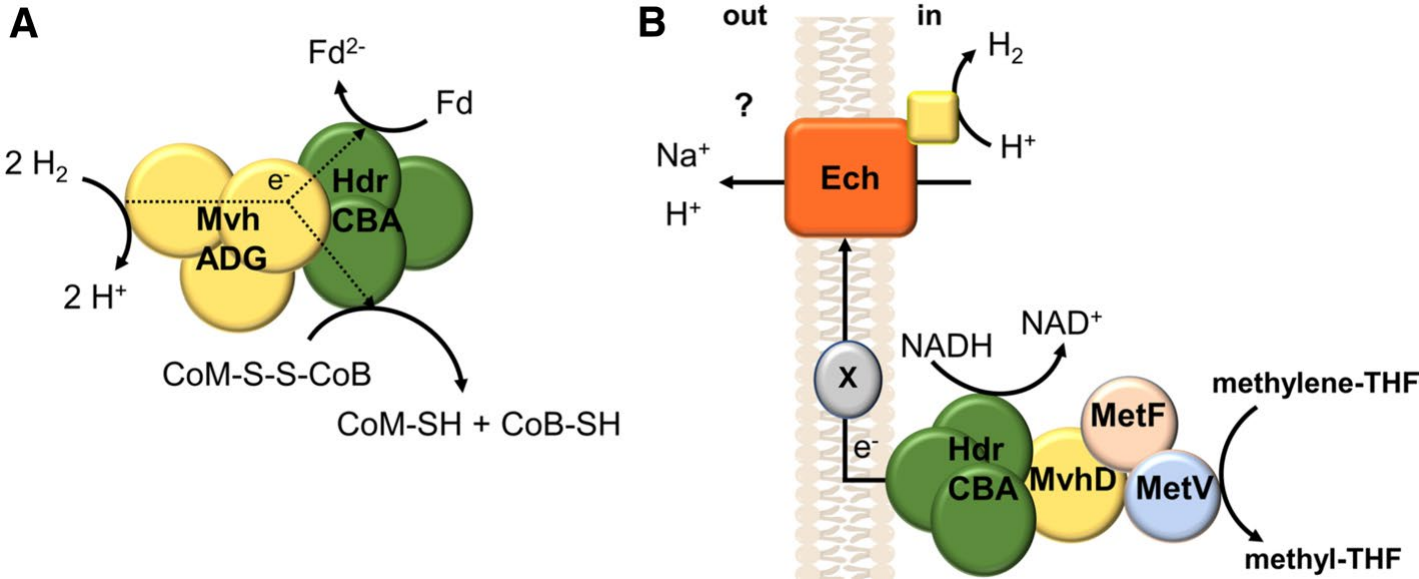
The electron-bifurcating heterodisulfide reductase of non-cytochrome-containing methanogens (**A**) and the possible electron-bifurcating methylene-THF reductase in acetogenic bacteria (**B**). Also depicted in panel B is a possible final electron acceptor that is oxidized by the Ech complex to reduce H^+^ to H_2_. The redox potential of known cytochromes and menaquinone would be too high (−200 and −74 mV) therefore, electron transfer may involve an unknown membrane-integral carrier (X). In this example, the MetFVHdrABCMvhD complex is assumed to bifurcate electrons from NADH to methylene-THF and finally to protons

Let us now turn to the MTHFR. Since this is the only exergonic reaction of the WLP and since methanogens had been shown by one of us already in 1988 to couple reduction of formaldehyde (methylene-THSP) to the methyl level with translocation of sodium ions across the membrane (Müller et al. 1988b), a rush started to identify the MTHFR as membrane-bound, electron transfer-driven ion pump. However, all attempts using *A. woodii*, *Acetobacterium dehalogenans*, *S. sphaeroides* and *M. thermoacetica* failed in a sense that most of the enzyme was always found in the soluble fraction, strongly arguing against a role in membrane-bound electron transport. Therefore, the enzyme fell into oblivion for decades. With the unraveling of genome sequences evidence for membrane localization was also not obtained and the view of a soluble MTHFR was solidified. Therefore, it was no surprise that the enzyme was purified from the cytoplasm of *A. woodii* (Bertsch et al. 2015). It contained the subunits MetF and MetV and an additional subunit, RnfC2, that catalyzes NADH oxidation. And indeed, the soluble enzyme catalyzed MTHF reduction with NADH as reductant (Bertsch et al. 2015). But again, the beauty of metabolic diversity gets into our way: many acetogenic species have *metFV* but not the *rnfC2* gene (Öppinger et al. 2021). Mock et al. described in *M. thermoacetica* a gene cluster that contains *metFV* next to *hdrCBA* and *mvhD*; these genes were part of one transcriptional message arguing for the proteins being functional in the same context (Mock et al. 2014). In the thermophile *Methanobacterium thermoautotrophicus*, the *hdr* genes were found to build an electron-bifurcating enzyme complex (Fig. 4A). H_2_ is oxidized by MvhA and the electrons are passed via MvhG and D to HdrA. The flavin in HdrA is the branching point, leading to the heterodisulfide- and ferredoxin-reduction site (Kaster et al. 2011; Wagner et al. 2017). Therefore, it is tempting to speculate that the HdrABCMvhD subunits confers electron bifurcation to the MTHFR. Although one has to consider important functional differences. HdrA of *M. thermoacetica* but not *M. thermoautotrophica* has a diaphorase activity oxidizing NADH and the HdrABCMvhDMetFV complex purified from *M. thermoacetica* does not reduce ferredoxin (Mock et al. 2014). A possible electron bifurcation by the complex could not be demonstrated, although several obvious electron carriers were tested. Therefore, the authors concluded that the complex “is somehow docked” to the membrane and the membrane respiratory electron acceptor has a redox potential comparable to ferredoxin, allowing for proton reduction to H_2_, as catalyzed by Ech (Fig. 4B).

Ech complexes often interact with carbon monoxide and, consistent with this hypothesis, CO- (reduced ferredoxin-) dependent electron transport at membrane vesicles of *M. thermoautotrophica* and the concomitant built up of a membrane potential has been observed (Hugenholtz and Ljungdahl 1989). A similar genetic organization of *metFVhdrCBAmvhD* was found in *T. phaeum* and also there the proteins were discussed to electrically connect the MTHFR to the membrane. In sum, it is conceivable that in some species (that have a MetFVHdrABCMvhD complex) the MTHFR is indeed electrically connected to the membrane. During carbon flow from CO_2_/formate, it serves as electron acceptor for a membrane-bound, energy-conserving respiratory chain that involves quinones and cytochromes, but the latter are not mandatory. If a NiFe hydrogenase is involved as electron input/output module, cytochrome *b* maybe involved. If a formate dehydrogenase is involved cytochromes are most likely not involved.

**Fig. 5:**
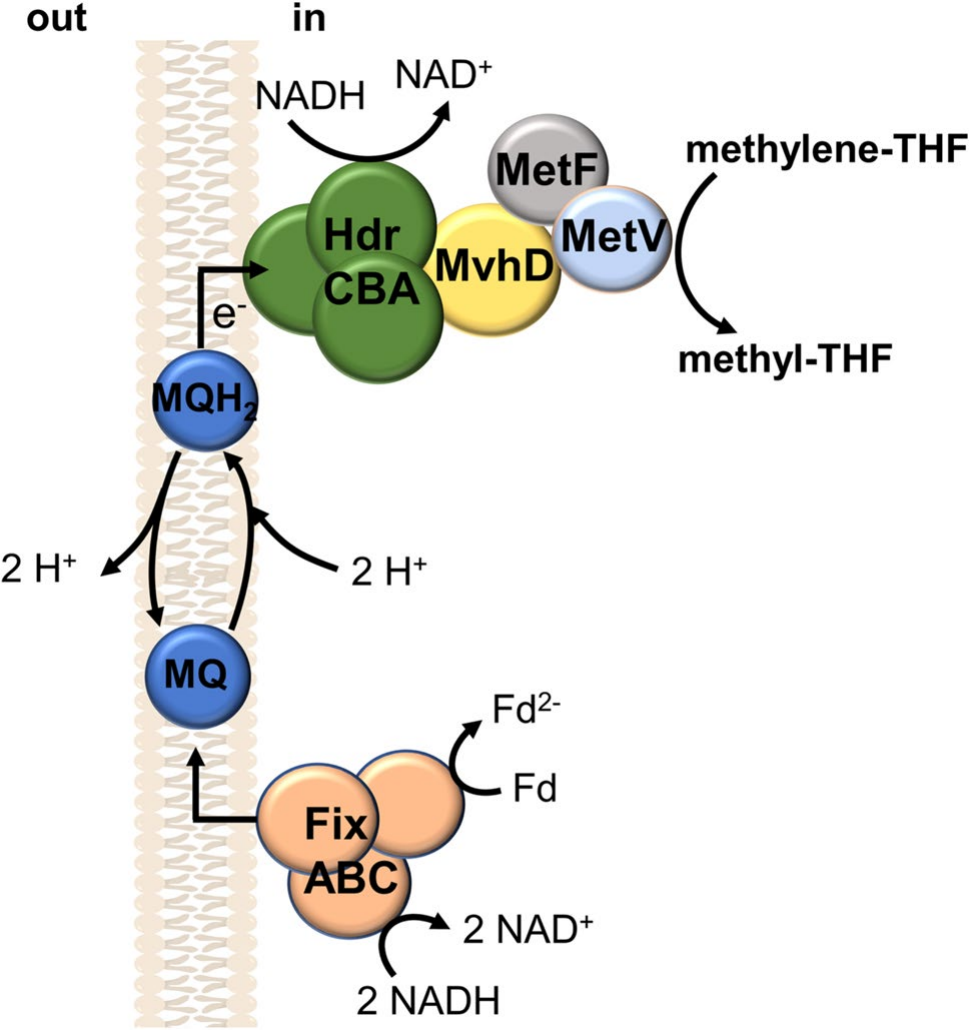
Hypothetical coupling of NADH oxidation to the reduction of methylene-THF by two consecutive electron-bifurcating reactions, catalyzed by FixABCX and the MetFVHdrABCMvhD complex. A quinone cycle may be involved in generating an electrochemical field across the membrane for ATP synthesis. Please note that proton translocation is related to oxidation/reduction of MQ and requires a membrane-integrated protein to catalyze this reaction

So far, we have discussed the role of cytochromes and quinones in electron transport processes in which the electron source such as formate or hydrogen is directly oxidized at the membrane. What about NADH as electron donor for quinone reduction? We already know about the beauty of metabolic diversity in acetogens and would wonder if there is no solution to involve NADH-dependent quinone reduction. Indeed, there is a solution to couple NADH oxidation and subsequent (mena)quinone reduction. Ledbetter and colleagues characterized a bifurcating enzyme complex called FixABCX, which has been proposed to oxidize NADH and reduce ferredoxin subsequently with quinone via bifurcation (Ledbetter et al. 2017). In this context, the role of FixABCX in acetogens has not yet been addressed. FixAB is equivalent to EtfAB, FixC is the quinone reductase. Interestingly, genes encoding FixABCX are present in only some acetogens such as *Moorella* and *Sporomusa* species (Table 1). NADH oxidation would allow for ferredoxin reduction, and the resulting high potential menaquinone could be the electron donor for a second bifurcating reaction, that uses NADH as co-reductant to drive the endergonic reduction of methylene-THF with the high potential menaquinone as electron donor (Fig. 5). In this context it is interesting to note that the FixABCX complex is widespread in bacteria and was recently shown to allow sulfate reducers to grow on ethanol. Oxidation of ethanol to acetaldehyde (E_0_’ = -190 mV) with reduction of NAD^+^ is endergonic and overcome by coupling to NADH oxidation by the electron-bifurcating FixABCDHdrABC complex (Ramos et al. 2015). Similarly, oxidation of lactate to pyruvate (E_0_’ = -190 mV) also does not allow for NAD^+^ reduction and acetogens have evolved different ways to overcome the energetic barrier. One is by electron bifurcation with concomitant oxidation of reduced ferredoxin (Weghoff et al. 2015), another recently suggests coupling to a FixABCX/membrane-bound electron transfer as predicted in Fig. 5 (Rosenbaum et al. 2021).

Since carbon dioxide does not serve exclusively as final electron acceptor in acetogens the question arises whether the reduction of alternative final electron acceptors may require quinones? In theory the answer is yes, the genome sequencing of *M. thermoacetica* reveals the presence of a DMSO reductase (Pierce et al. 2008). Since there is no experimental evidence for DMSO reduction by acetogens, it can be only speculated whether and if so, how DMSO is reduced in this ecophysiologically important group of bacteria.

## Conclusions

Cytochromes and quinones were the first respiratory components found in acetogens. Unfortunately, they sank into a long sleeping beauty slumber, which is not yet really over. Cytochrome-dependent respiratory chains in acetogens are suggested for nearly 50 years but it is far from being settled whether they are present or not. However, if they are present, they are only in addition to the Rnf or Ech complex. Here, the situation is comparable to methanogens. Every methanogen that grows on H_2_ + CO_2_ and that has been studied or sequenced so far has the sodium ion pump methyl-THMP/THSP:CoM methyltransferase, but some have cytochrome-containing respiratory chains in addition. Every acetogen possesses an energy coupling site, either Rnf or Ech but just a few acetogens have in addition cytochromes and quinones, such as *Sporomusa*, *M. thermoacetica* or *Clostridium aceticum* (Gottwald et al. 1975; Poehlein et al. 2015; Kamlage et al. 1993). Recently, the presence of a Na^+^-active electron transport phosphorylation with Na^+^-Rnf and Na^+^-ATP synthase was described in *C. aceticum*, cytochromes are present in addition but of unknown function (Wiechmann and Müller 2021) Important for the context here is the notice that the strains that are currently used in industrial applications do not have cytochromes or quinones.

Now it is time to wake up the cytochromes from sleep. Genetic techniques have been developed for some acetogens, including the cytochrome-containing acetogen *M. thermoacetica;* for other model organisms such as *S. ovata,* a genetic system is missing. These genetic systems need to be established, to prove or disprove the postulated electron transport chains. In addition, classical biochemical techniques such as the preparation of intact inverted membrane vesicles need to be established for these species. Anyway, the time is ripe to address the role of cytochromes and quinones in acetogens, nearly 50 years after their discovery. The beauty of microbial diversity will certainly increase and with this the fascinating diversity of microbial physiology.

## Abbreviations

WLP: Wood–Ljungdahl pathway
THF: Tetrahydrofolate
CODH/ACS: CO dehydrogenase/acetyl-CoA synthase
Coenzyme M: 2-Mercaptoethanesulfonate
Coenzyme B: 7-Mercaptoheptanoylthreoninephosphate
Ech: Energy converting hydrogenase
Rnf: *Rhodobacter* Nitrogen fixation
CoFeSP: Corrinoid/iron sulfur protein
BV: Benzylviologen
MF: Methanofuran
MQ-7: Menaquinone-7 (2-methyl-3-heptaprenyl-1.4-naphtoquinone)
MTHF: Methylene-THF
MTHFR: Methylene-THF reductase
[H]: Reducing equivalent, one electron
Cyt: Cytochrome
MP: Methanophenazine
F_420_: Coenzyme F_420_
F_420_H_2_: Reduced coenzyme F_420_
Frh: Coenzyme F_420_ reducing hydrogenase
FDH: Formate dehydrogenase
UQ: Ubiquinone
